# Intrauterine oxygen milieu governs placental sphingolipid metabolism

**DOI:** 10.1016/j.jlr.2025.100930

**Published:** 2025-10-28

**Authors:** Julien Sallais, Martin Post, Isabella Caniggia

**Affiliations:** 1 Lunenfeld-Tanenbaum Research Institute, Sinai Health System, Toronto, Ontario, Canada; 2 Institute of Medical Sciences, University of Toronto, Toronto, Ontario, Canada; 3 Program in Translational Medicine, Peter Gilgan Learning Centre for Research and Learning, The Hospital for Sick Children, Toronto, Ontario, Canada; 4 Departments of Obstetrics & Gynaecology, University of Toronto, Toronto, Ontario, Canada

**Keywords:** Placenta, trophoblast, oxygen, sphingolipids, mitochondrial dynamics

## Abstract

Early placentation relies on temporal changes in intrauterine oxygen tension that regulate trophoblast differentiation events. Studies have highlighted the contribution of bioactive sphingolipids to the pathogenesis of placental disorders, characterized by hypoxia. However, it is unknown whether placental sphingolipid metabolism changes during the switch from a hypoxic to an oxygenated environment in the first trimester of gestation and if sustained hypoxia is causative of sphingolipid alterations seen in preeclampsia. Herein, we performed sphingolipid analysis of first-trimester human placentae as well as placentae from conditional (placenta-specific) *Phd2* knockout mice (*Phd2*^−/−^ cKO) that exhibit preeclampsia-like features, including placental hypoxia. Analysis revealed elevated long chain ceramide (Cer16:0, Cer18:0, Cer20:0 and Cer22:0) and reduced sphingosine-1-phosphate (So-1-P) content in *Phd2*^−/−^ cKO placentae. Expression of key regulatory sphingolipid enzymes, acid ceramidase (ASAH1) and sphingosine kinase 1 (SPHK1), was reduced in *Phd2*^*−/−*^ cKO placentae, while that of alkaline ceramidase ACER2 remained unchanged. Human placentae from 5-9 weeks of gestation, when intrauterine oxygen tension is low, exhibited heightened long chain ceramide (Cer14:0, Cer16:0, Cer18:0, Cer 18:1) and sphingosine content and reduced ASAH1 and SPHK1 expression, highlighting the relevance of low oxygen in regulating sphingolipid metabolism under physiological (placental development) and pathological (*Phd2*^−/−^ cKO induced preeclampsia) conditions. Ultrastructural analyses of early (5–9 weeks) human and murine *Phd2*^−/−^ cKO placentae revealed that increased trophoblast mitochondrial fission events accompanied elevated ceramide. Together, the data support the concept that a chronic low-oxygen environment leads to placental ceramide buildup, which may alter mitochondrial homeostasis and potentially contribute to cell death events characteristic of preeclampsia.

Early placentation is a dynamic and tightly regulated process that relies on temporal changes in intrauterine oxygen tension (1, 2, 3). During the initial stages of implantation, both the embryo and developing placenta are exposed to a relatively hypoxic (pO_2_ < 20 mmHg) environment (2, 4). The low oxygen tension serves as a signal for the differentiation and migration of trophoblast cells towards the maternal decidua (2, 5). Subsequently, around 10–12 weeks of gestation, a physiological shift occurs as the intervillous space opens, exposing the developing placenta to oxygenated maternal blood, leading to an increase in placental oxygenation to ∼60–80 mmHg (2, 4). This sudden surge in oxygen availability triggers a transition from a proliferative to an invasive trophoblast phenotype, facilitating the remodeling of maternal spiral arteries to ensure adequate supply of oxygen and nutrients to the developing fetus (5, 6). Hypoxia Inducible Factor (HIF)1A plays a crucial role in regulating these trophoblast differentiation events (7, 8). The stability of HIF1A is mediated by a family of oxygen-sensitive prolyl hydroxylases (PHD) (7, 9, 10). By sensing physiological variations in intrauterine oxygen tension, the PHD/HIF1A axis orchestrates the expression of multiple HIF1A-regulated pathways that influence trophoblast cell fate and, consequently, proper placental organogenesis (3, 11, 12). Disruption of this crucial axis results in persistent HIF1A levels, leading to placental pathologies such as preeclampsia (12, 13, 14).

Sphingolipids have emerged as important signaling and regulatory molecules within various biological systems, playing vital roles in a wide range of physiological and pathological processes, including cell proliferation, migration, angiogenesis, inflammation and cell death (15, 16, 17, 18). Among the sphingolipid species, ceramide is at the core of sphingolipid metabolism, serving as a precursor for all other sphingolipids (16, 18). It is generated through three primary pathways: 1) de novo synthesis from serine and palmitoyl coenzyme A via serine palmitoyltransferase, 2) breakdown of sphingomyelin by acid or neutral sphingomyelinases, and 3) conversion from sphingosine by ceramide synthase. Ceramides can be deacylated by ceramidases into sphingosine that then can be converted into sphingosine-1-phosphate (So-1-P) through the action of sphingosine kinase or acetylated by ceramide synthase in the salvage pathway to produce ceramide, highlighting the dynamic nature of sphingolipid metabolism (15, 19). Studies from our laboratory and others have highlighted the consequences of ceramide accumulation in the placenta, including triggering trophoblast cell death via autophagy (20) and necroptosis (21), provoking endothelial dysfunction (22) and shifting trophoblast mitochondrial homeostasis toward fission in preeclampsia (20, 23) and gestational diabetes mellitus (24, 25, 26). Despite this growing body of evidence linking sphingolipids to pregnancy-related disorders, particularly in response to oxidative stress, placental sphingolipid metabolism and mitochondrial homeostasis during early placental development, and their response to intrauterine variations in physiological oxygen levels remain unknown.

Here, we examined how changes in physiological oxygen tension during the first trimester of gestation (ranging from pO_2_ < 20 mmHg at 5–9 weeks to 60–80 mmHg at 10–15 weeks of gestation) impact human placental sphingolipid metabolism and associated mitochondrial homeostasis. We observed significant differences in ceramide levels and mitochondrial fission events during the intrauterine switch in oxygen tension, and investigations in mice with a placental knockout of *Phd2* revealed that a low oxygen environment is directly responsible for these changes.

## Materials and methods

### Animals

All procedures involving animals were performed in compliance with the Animals for Research Act of Ontario and the Guidelines of the Canadian Council on Animal Care and were approved by The Centre for Phenogenomics (TCP) Animal Care Committee (AUP#19–0286). Placental*-*specific *Phd2*^*−/−*^ embryos were generated as previously described (27). Briefly, *Tpbpa*^*cre*^ mice were bred with *Phd2*^*flox/flox*^ mice (both mice were maintained on a C57BL6 background) to produce *Tpbpa*^*cre*^*;Phd2*^*flox/flox*^ mice*. H*omozygous *Tpbpa*^*cre*^*;Phd2*^*flox/flox*^ males were then selected to mate with female *Phd2*^flox/flox^ mice to generate placental*-*specific *Phd2*^*−/−*^ cKO embryos, while control WT embryos were produced by breeding male *Phd2*^*flox/flox*^ mice with female *Phd2*^*flox/flox*^. Pregnant animals were sacrificed at E14.5 and E17.5 using CO_2_ euthanasia. Upon sacrifice, dissection was performed, placentae were collected, weighed and fixed in paraformaldehyde or snap frozen for further analysis.

### Human tissue collection

Informed consent was obtained from pregnant individuals in accordance with the Ethics Guidelines of the University of Toronto’s Faculty of Medicine and Mount Sinai Hospital, Toronto. Placental tissue from early gestation, 5–15 weeks (n = 24) were collected from elective terminations of pregnancies by dilation and curettage or suction evacuation. The Research Center for Women and Infant Health (RCWIH) Biobank from Mount Sinai Hospital performed all the collections following standardized operating procedures that can be found online on the RCWIH website (http://biobank.lunenfeld.ca). All human studies reported herein were conducted in accordance with the principles of the Declaration of Helsinki. Immediately following collection, human samples were fixed and/or snap frozen and stored appropriately for further processing.

### Sphingolipid analyses

Placental tissues were collected and kept at −80°C until lipid extraction. Twenty-five mg of frozen tissue was lyophilized, transferred to siliconized conical glass tubes and homogenized in 2 ml of (1:1) methanol/water. Samples were spiked with a mixture of internal C17-based standards [(d18:1/17:0) ceramide, (d17:1) sphingosine, (d17:0) sphinganine-1-phosphate, (d17:1) sphingosine-1-phosphate and (d18:1/17:0) sphingomyelin; 10 ng of each (Avant Polar Lipids, Alabaster, AL)]. After the addition of 2 ml of chloroform, samples were vortexed for 1 min, kept on ice for 10 min, and then centrifuged at 1,000*g* for 5 min. The chloroform layer was transferred to a new set of siliconized conical glass tubes. The remaining fraction was then acidified with 40 μL of 0.1 N HCl, and another 1 ml methanol was added, followed by 2 ml of chloroform. Again, samples were vortexed for 1 min, kept on ice for 10 min and then centrifuged at 1,000*g* for 5 min. The chloroform layer was collected and combined with the previous extract and dried under a stream of nitrogen. Samples were then reconstituted in 100 μl ethanol acidified with 2 μl of formic acid. Samples were split and transferred to siliconized minivials for analysis by liquid chromatography tandem mass spectrometry (LC-MS/MS). For ceramide (Cer) and sphingomyelin (SM) measurements, 10 μl of the reconstituted sample was diluted 1:10 in ethanol/formic acid solution. For sphingosine-1-phosphate (S-1-P), sphingosine (So), and sphinganine (Sa) measurements, 20 μl of water containing 0.2% formic acid was added to the remaining 90 μl of reconstituted sample. LC-MS/MS for sphingolipids was performed on an Agilent 1290 Series binary pump (Agilent Technologies Inc) coupled to an AB5500 triple-quadrupole mass spectrometer (Sciex, Concord, ON, Canada). For Cer and SM analysis, reverse phase high-performance liquid chromatography (HPLC) was performed using a Kinetex C18 column (2.6 μm, 100 × 2.1 mm: Phenomenex). The sample injection volume was 5 μl. The mobile phase consisted of (A) water/acetonitrile/methanol (2/1/1, v/v/v) and (B) tetrahydrofuran/acetonitrile/methanol (2/1/1,v/v/v) with both components containing 0.05% formic acid. The HPLC gradient was as follows: T = 0–4 min 40% B, flow rate 350 μl/min; T = 4–7 min 60% B, flow rate 350 μl/min; T = 7–11 min 70% B, flow rate 400 μl/min; T = 11–13 min 85% B, flow rate 400 μl/min; T = 13–19 min 40% B, flow rate 350 μl/min. For S-1P, So and Sa analysis, samples (6 μl) were injected onto a Kinetex XB-C18 column (2.6 μm, 50 × 3.0 mm: Phenomenex Torrance). Samples were eluted using a gradient consisting of (A) water containing 0.2% formic acid and (B) tetrahydrofuran/acetonitrile/methanol (2/1/1, v/v/v) containing 0.1% formic acid. At a flow rate of 600 μl/min, the HPLC gradient was as follows: T = 0–0.5 min 50% B, T = 0.5–2.0 min 80% B, T = 2.0–4.5 min 98% B, T = 4.5–6 min 50% B. MS was performed in positive electrospray ionization mode. The source temperature was maintained at 400°C with the ion spray voltage set at 5,000 V and nitrogen used as the Collision Induced Dissociation gas. MRM Mass Transitions and Chromatographic Retention Times of sphingolipids are shown in Table 1. Although dihydrosphingomyelin species were included in the targeted LC-MS/MS panel, no detectable levels were observed in any of the experimental samples and thus were excluded from further analysis. A separate standard curve was generated for each analyte measured using MRM area ratios for quantitative analysis (Analyte Peak Area/IS Peak Area), and the results were then calculated by plotting the sample area ratios against their respective analyte-specific standard curve. Data integration and quantitation were performed using AB Sciex Analyst 1.6 software.

**Table 1:** Sphingolipid MRM mass transitions and chromatic retention time

Sphingolipid	MRM mass transition (Da)	Collision energy (eV)	Retention time (min)
(IS) So (d17:1)	286.4/268.1	23	1.46
So (d18:1)	300.4/282.1	23	1.57
(IS) Sa1P (d17:1)	368.4/270.2	30	1.42
Sa (d18:1)	302.4/284.1	23	1.62
(IS) So1P (d17:1)	366.4/250.1	30	1.36
So1P (d18:1)	380.4/264.2	30	1.47
SM 12:0	647.7/184.1	40	5.54
DiHySM 12:0	649.7/184.1	40	5.88
SM 16:0	703.7/184.1	40	7.33
SM 18:1	729.7/184.1	40	7.48
(IS) SM 17:0	717.7/184.1	40	7.80
CER 14:0	510.6/264.4	40	8.06
SM 18:0	731.7/184.1	40	8.26
CER 16:0	538.6/264.4	40	9.38
Cer 18:1	564.6/264.4	30	9.62
DiHyCer 18:1	566.6/284.5	30	10.16
(IS) CER 17:0	552.5/264.4	60	10.14
CER 18:0	566.6/264.4	40	10.69
SM 24:1	813.7/184.5	40	10.64
DiHyCer 18:0	568.8/284.5	30	10.98
SM 24:0	815.6/184.1	40	11.41
CER 20:4	594.6/264.4	40	11.41
CER 24:1	648.6/264.4	40	11.86
CER 22:0	622.6/264.4	40	11.93
DiHyCer 24:1	650.7/284.5	30	12.04
DiHyCer 24:0	652.7/284.5	30	12.59

### Quantitative PCR

Total RNA was extracted from 5-10 mg of frozen placental tissues using RNeasy® Plus Mini Kit (Qiagen). One μg of total RNA was reverse transcribed using qScript cDNA Synthesis Kit (Quanta Biosciences). The resulting cDNA were quantified by real-time PCR (CFX96 Real-Time System, Biorad) using PerfeCTa FastMix II from Quantabio (Beverly) and mouse specific TaqMan® (Assays-on-Demand™) probes targeting *18s* (Mm03928990_g1), *Asah1* (Hs00602774_m1), Mm00480021_m1, *Sphk1* (Hs00184211_m1, Mm00448841_g1), and *Acer2* (Hs04996319_g1, Mm00519876_m1) from Applied Biosystems (ThermoFisher Scientific). For each probe, a dilution series determined the efficiency of amplification of each primer set. Gene expression was expressed as the relative fold change compared to the expression of the small subunit 18s ribosomal RNA, using the delta-delta Ct method, and compared to selected appropriate positive or negative controls.

### Western blotting

Flash frozen tissue was crushed, dissolved in RIPA buffer containing protease inhibitors and homogenized at 4°C to generate placentae tissue lysates that were centrifuged at 14,000*g* for 10 min at 4°C. The supernatant was collected, and protein concentration was quantified using Bradford protein assay (Bio-Rad) For Western blotting, protein lysates (ranging from 25 to 200 μg of protein) were subjected to SDS-PAGE on 7.5% (SPHK1 protein analysis), 10% (SPHK2, ASAH1and SMPD1 protein analysis) and 12% (ACER2 protein analysis) acrylamide gels (Bio-Rad). After each run, the gels were imaged for a stain-free profile of total protein using the BioRad Chemidoc XRS+ System. Subsequently, separated proteins were transferred to polyvinylidene fluoride (PVDF) membranes, incubated for 1 h in either 5% non-fat dry milk or 5% bovine serum albumin (BSA) diluted in Tris-buffered saline containing Tween-20 (0.1% vol/vol, (TBS-T) and then probed overnight at 4°C with primary antibodies diluted in the corresponding blocking buffer. The next day, membranes were washed in TBS-T and incubated with appropriate secondary HRP (horseradish peroxidase)-conjugated antibodies for 1 h at room temperature. Membranes were washed with TBS-T, and immunoreactivity was visualized following the addition of chemiluminescence ECL reagent (Bio-Rad®, Mississauga, Ontario) and equal exposure time using Bio-Rad Chemidoc XRS+ System. Densitometric analysis was performed using ImageLab software with data normalized to either total protein in stain free gels or ACTB (β-actin) protein. Antibody source and dilutions are shown in Table 2.

**Table 2:** Antibody source and dilutions

Name	Company	Catalog #	Clonality	Dilutions
ACER2	Invitrogen	PA5-101415	polyclonal	WB [1:1000]
ACTB	Santa Cruz Biotechnology	sc-47778	monoclonal	WB [1:2000]
ASAH1	Santa Cruz Biotechnology	sc-28486	polyclonal	WB [1:500], IF [1:200]
ASAH1	Aviva System Biology	OAPB00726	polyclonal	WB [1:1000], IHC [1:250]
OPA1	Abcam Inc	ab157457	monoclonal	WB [1:1000]
pDRP1	Cell signaling Technology	3455s	polyclonal	WB [1:700]
SMPD1	Santa Cruz Biotechnology	NBP2-22365	polyclonal	WB [1:500]
SPHK1	Abcam Inc	ab260073	polyclonal	WB [1:1000]
SPHK2	Santa Cruz Biotechnology	sc-22704	polyclonal	WB [1:500]

### Immunohistochemistry

Immediately following collection, placentae were fixed in 4% (v/v) paraformaldehyde for 24 h. Samples were then dehydrated, embedded in paraffin and sectioned using a Leica RM2255 (Leica Biosystems, Concord, Ontario, Canada) microtome. For immunohistochemical staining, 5-micron sections were deparaffinized and rehydrated. Following antigen retrieval (10 mM sodium citrate, pH 6.0), sections were treated with 3% (v/v) H_2_O_2_ in methanol to block endogenous peroxidase activity, washed in PBS and incubated for one hour with blocking solution (5% (v/v) normal horse serum and 1% (v/v) BSA in PBS). After washing, sections were probed with primary antibody diluted in blocking solution overnight at 4°C followed by biotinylated secondary antibody for 2 h at room temperature. Sections were washed, incubated for one hour with avidin/biotin-horseperoxidase complex (VectaStain ABC Standard Kit; Vector Laboratories), and formed complexes were identified with DAB (3,3′-diaminobenzidine) substrate (0.075% [w/v] DAB in PBS containing 0.002% [v/v] H_2_O_2_). Sections were counterstained with hematoxylin and mounted with Surgipath micromount medium (Leica). Images were captured using an Olympus BX61 motorized light microscope system. Antibody source and dilutions are shown in Table 2.

### Transmission electron microscopy

Placentae were fixed in 2% (v/v) glutaraldehyde in 0.1 M cacodylate buffer (pH 7.3) for up to 24 h at 4°C. Samples were processed by the Nanoscale Biomedical Imaging Facility at The Hospital for Sick Children, Toronto. Samples were rinsed in buffer, then post-fixed in 1% osmium tetroxide buffer. Following dehydration using an ascending series of ethanol (50%, 70%, 90%, and 100%), samples were embedded in Quetol-Spurr resin. Leica EM UC7 ultramicrotome was used to produce 80 nm sections that were subsequently stained with uranyl acetate and lead citrate and deposited on copper grids. TEM Images were captured with a FEI Technai 20 electron microscope (FEI). Mitochondria were identified by their characteristic shape and cristae content. Mitochondrial perimeter, diameter, ferret’s diameter (longest distance between two points within a given mitochondrion) and surface area were measured as previously described (20).

### Statistical analysis

All analyses were performed using MetaboAnalyst 5.0, a widely used web-based platform for comprehensive metabolomics data analysis (28).

#### Data preprocessing

Each of the three independent datasets (early-gestation human placentas, murine placentae at E14.5, and murine placentae at E17.5) was processed and analyzed separately to account for biological differences. Prior to statistical analysis, raw sphingolipid concentration data were normalized and scaled. For the human and E14.5 mouse datasets, normalization by sum (i.e. each sample’s values were scaled so that the sum of all measured sphingolipid abundances was identical across samples) was applied, followed by a log10 transformation and autoscaling. Logarithmic transformation was used to correct for heteroscedasticity and to make right-skewed distributions more symmetric (29). Autoscaling (unit variance scaling) was then performed by mean-centering each variable and dividing by its standard deviation, giving all sphingolipid species a standard deviation of 1 (29). This approach places abundant and less-abundant lipids on a comparable scale and focuses the analysis on relative differences rather than absolute concentration magnitudes. The E17.5 mouse dataset did not require log transformation (as its value distributions were approximately normal). For that dataset, sum-normalized values were used directly (with autoscaling applied) in subsequent analyses.

#### Sparse Partial Least Squares Discriminant Analysis (sPLS-DA)

Multivariate analysis of the sphingolipid profiles was carried out using sparse Partial Least Squares Discriminant Analysis (sPLSDA) (30). This supervised multivariate technique was chosen to assess overall differences in sphingolipid composition between experimental groups (e.g. 5–9 weeks vs. 10–15 weeks and *Phd2*^*−/−*^ cKO vs. WT genotypes) while performing integrated feature selection. Separate sPLS-DA models were built for each dataset in MetaboAnalyst. In each model, samples were projected into a new coordinate space defined by latent components that maximize the separation between the two groups, with an L1 penalty applied to encourage sparsity (*i.e*., selection of a subset of informative lipid variables) (30). The primary output was a two-dimensional score plot for each dataset, showing individual placenta samples plotted by their scores on the first two sPLS-DA components. These score plots were examined for clustering or separation of samples according to genotype. To aid interpretation, 95% confidence ellipses were drawn around the clusters for each group in the score plots, illustrating the multivariate spread of each group. In addition to sample scores, the sPLS-DA provided loading values and Variable Importance in Projection (VIP) scores for each sphingolipid species. These metrics quantify the contribution of each lipid to group discrimination. Variables with higher VIP scores were deemed more influential in driving the separation between cKO and WT groups (31).

#### Heatmap visualization

To visualize the global patterns of sphingolipid content in each dataset, we generated heatmaps of the normalized lipid data using MetaboAnalyst’s heatmap function. Each heatmap included all quantified sphingolipid species as rows, and individual samples as columns, grouped by genotype. A color gradient was used to represent the relative abundance of each lipid. For clarity, no hierarchical clustering was applied to these heatmaps. Lipid species were instead ordered by category (*e.g*., ceramides, sphingomyelin and sphingosine). The heatmaps provided an overview of how all measured sphingolipids differed in their abundance profiles between experimental groups in each dataset.

#### Volcano plot analysis (lipid-specific testing)

To identify differentially abundant sphingolipid species between experimental groups, volcano plots were generated using the Volcano Plot module in MetaboAnalyst 5.0. This approach integrates fold change (FC) analysis and non-parametric hypothesis testing to highlight features of interest based on both statistical significance and magnitude of change. Sphingolipid datasets from human placenta and murine placenta at gestational days E14.5 and E17.5 were each analyzed independently. Given the non-normal distribution observed in approximately 50% of sphingolipid species across datasets (Shapiro-Wilk test, data not shown), a non-parametric Wilcoxon rank-sum test (Mann–Whitney U test) was applied. Analyses were conducted in unpaired mode, assuming unequal group variances, and using a raw *P*-value threshold of 0.05 for significance. Volcano plots were generated by plotting the log2 fold change (log2FC) on the x-axis against the negative log10-transformed *P*-value on the y-axis. A fold-change threshold of 1 (equivalent to 2-fold difference on the linear scale) was used to distinguish meaningful biological shifts. Fold change was calculated from original concentrations (i.e., before transformation), whereas *P*-values reflect testing on the transformed data. Fold Change and *P*-values were reported for each figure in their respective table (Table 3, Table 4, Table 5).

**Table 3:** Supporting table for volcano plot of human placental development shown in Fig. 1

Significant Species	FC	log2 (FC)	Raw *P*-value	−LOG10 (*P*-value)
Cer 14:0	0.46584	−1.1021	7.17E-05	4.1442
Cer 16:0	0.57058	−0.80951	0.000371	3.4303
Cer 18:1	0.62066	−0.68813	0.001115	2.9529
SM 12:0	0.67354	−0.57016	0.008293	2.0813
Sa	0.43514	−1.2005	0.024184	1.6165
So	0.58554	−0.77216	0.024184	1.6165

*P*-value was obtained using a Wilcoxon Rank test.

FC, Fold Change.

**Table 4:** Supporting table for volcano plot of mouse placenta samples at E14.5 in Fig. 3

Significant Species	FC	log2 (FC)	Raw *P*-value	−LOG10 (*P*-value)
SM 24:1	0.7751	−0.36754	0.002014	2.6959
SM 18:0	0.7633	−0.38967	0.01086	1.9642
Cer 22:0	1.2337	0.30296	0.016783	1.7751
Cer 24:0	1.2567	0.32965	0.028711	1.542

*P*-value was obtained using a Wilcoxon Rank test.

FC, Fold Change.

**Table 5:** Supporting table for volcano plot of mouse placenta samples at E17.5 show in Fig. 3

Significant Species	FC	log2 (FC)	Raw *P*-value	−LOG10 (*P*-value)
Sa	0.60841	−0.71688	1.47E-05	4.8327
Sa1P	0.57857	−0.78944	0.001434	2.8435
Cer 16:0	1.3428	0.42525	0.00224	2.6498
So1P	0.54924	−0.86448	0.004752	2.3232
Cer 18:0	1.2625	0.33627	0.004752	2.3232
SM 18:1	1.2255	0.29342	0.005146	2.2885
Cer 20:0	1.2808	0.357	0.008174	2.0875
SM 24:0	1.2854	0.36223	0.012656	1.8977
SM 24:1	1.2625	0.33629	0.01358	1.8671
SM 16:0	1.2102	0.27526	0.014562	1.8368
SM 18:0	1.2075	0.27202	0.014562	1.8368
Cer 22:0	1.206	0.27021	0.028217	1.5495

*P*-value was obtained using a Wilcoxon Rank test.

FC, Fold Change.

#### Individual lipid scatter plot visualization

For data visualization, we generated simple scatter plots for selected lipid species to illustrate the group differences. These plots were created using GraphPad Prism (version 9.5.0; GraphPad Software). The group comparisons and *P*-values from the Mann–Whitney U tests are annotated on the plots (*e.g*., a symbol for *P* < 0.05 or “ns” if not significant). These graphical representations complement the statistical analysis by visually conveying the magnitude and variability of differences in specific lipid levels between groups. All statistical methods were performed with two-tailed tests and a significance criterion of *P* < 0.05, as noted above.

## Results

### Ceramide metabolism and so-1-P levels during early human placental development

**Fig. 1:**
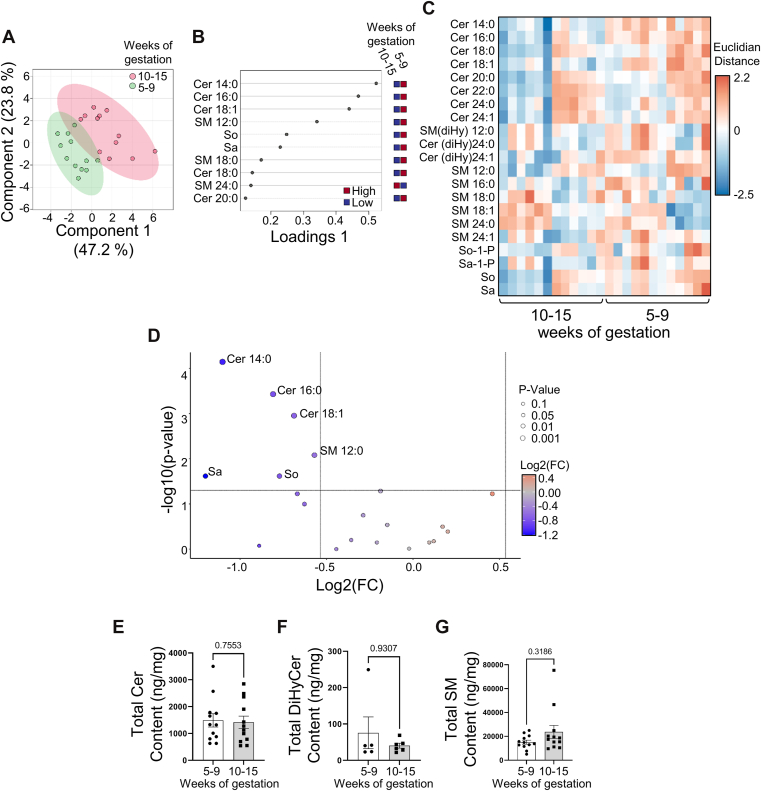
Long-chain ceramide and sphingosine content is elevated during early human placental development. A: Sparse Partial Least Discriminant Analysis (sPLS-DA) score plot showing the separation between sphingolipids in 5–9 (green) and 10–15 weeks (red) human placental tissue (5–9 weeks: n = 12 samples; 10–15 weeks: n = 12 samples). The colored circles represent the 95% confidence regions. B: Loading plots from sPLS-DA showing the top discriminating sphingolipid species contributing to the gestational group separation. C: heatmap of sphingolipid species abundance in human placental samples across early gestation, displayed without clustering. Each column represents an individual placenta (5–9 weeks: n = 12 samples; 10–15 weeks: n = 12 samples). Scaled abundance is color-coded from low (blue) to high (red). D: Volcano plot of sphingolipid species abundance of 10–15 weeks versus 5–9 weeks placentae. Reduced abundance was depicted in blue, while increased abundance is depicted in orange. Statistical Significance was evaluated using a Wilcoxon-test and fold-change threshold was set higher than 1.45 (5–9 weeks: n = 12 samples; 10–15 weeks: n = 12 samples). Only significant species (P > 0.05) are labelled. E–G: Scatter plot for total (E) ceramide (Cer), (F) dihydroceramide (DiHyCer) and (G) sphingomyelin (SM) content in human placental samples collected between 5 and 15 weeks of gestation (no statistical differences, non-parametric Mann–Whitney U test, Cer and SM 5–9 weeks: n = 12 samples; 10–15 weeks: n = 12 samples; DiHyCer species were sampled in 5–9 weeks: n = 5 samples; 10–15 weeks: n = 5 samples). w = weeks.

**Fig. 2:**
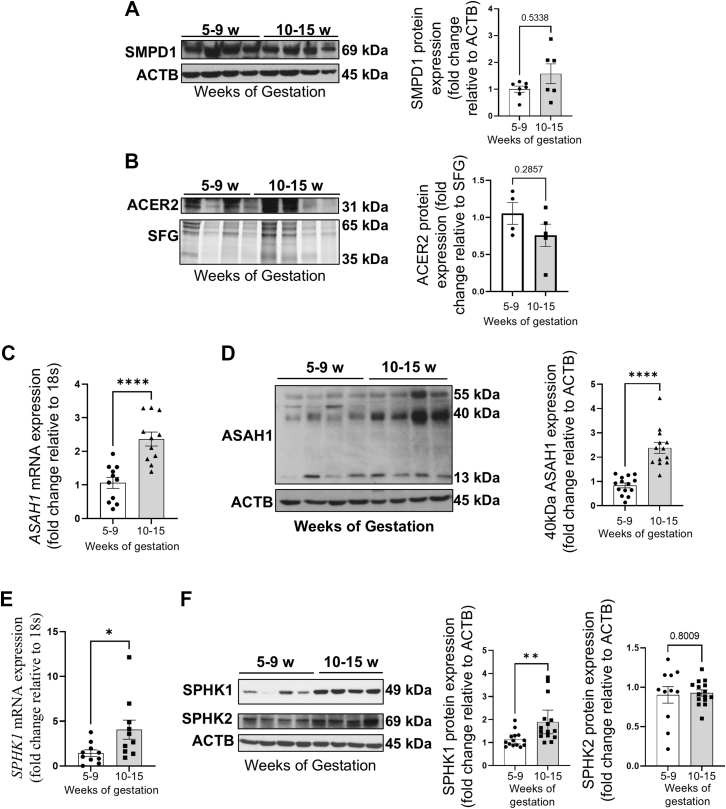
Expression of sphingolipid regulatory enzymes changes in early human placental development. A: representative immunoblots (left panel) and associated densitometry (right panel) for SMPD1 in human placental lysates collected between 5 and 15 weeks of gestation (no statistical differences, non-parametric Mann-Whitney test, 5–9 weeks: n = 7 samples; 10–15 weeks: n = 6 samples). B: representative immunoblots (left panel) and associated densitometry (right panel) for ACER2 in human placental lysates collected between 5 and 15 weeks of gestation (∗: *P* < 0.05, non-parametric Mann-Whitney test, 5–9 weeks: n = 4 samples; 10–15 weeks: n = 5 samples). C: Quantitative PCR for *ASAH1* mRNA in placental lysates collected between 5 and 15 weeks of gestation (∗∗∗∗: *P* < 0.0001, unpaired Student’s t test, 5–9 weeks: n = 11 samples; 10–15 weeks: n = 11 samples). D: representative immunoblots (left panel) and associated densitometry (right panel) for ASAH1 in human placental lysates collected between 5 and 15 weeks of gestation (∗∗∗∗: *P* < 0.0001, unpaired Student’s t test, 5–9 weeks: n = 14 samples; 10–15 weeks: n = 13 samples). E: Quantitative PCR for *SPHK1* mRNA in human placental lysates collected between 5 and 15 weeks of gestation (∗: *P* < 0.05, non-parametric Mann-Whitney test, 5–9 weeks: n = 10 samples; 10–15 weeks: n = 10 samples). F: representative immunoblots (left panel) and associated densitometry (right panel) for SPHK1 and SPHK2 in human placental lysates collected between 5 and 15 weeks of gestation (SPHK1: ∗∗: *P* < 0.01; SPHK2: no statistical significance, non-parametric Mann-Whitney test, 5–9 weeks: n = 14 samples; 10–15 weeks: n = 12 samples). w = weeks.

We conducted sphingolipid MS/MS analyses of human placentae collected during the first trimester of gestation. Samples were divided into two groups based on placental pO_2_ levels: 5–9 weeks, when pO_2_ is < 20 mmHg, and 10–15 weeks, when pO_2_ rises to 60 mmHg. We first evaluated global changes in placental sphingolipid composition across early gestation using sPLS-DA (Fig. 1A). The dataset included all the quantified sphingolipid species. The scores plot revealed a clear separation between the two gestational groups mostly along the first and second component (variance of 47.2% and 23.8%, respectively), with minimal overlap between samples clusters (Fig. 1A). To identify which lipid species contributed most to the observed separation, we examined the variable importance in projection (VIP) scores derived from component 1 (Fig. 1B). Among the top discriminating features were several ceramide species, including Cer14:0, Cer16:0, and Cer18:1, as well as sphingosine (So), sphinganine (Sa), and several sphingomyelin species including SM12:0, SM18:0 and SM24:0. To visualize the distribution of sphingolipids across all samples, a heatmap was generated using the full dataset of measured sphingolipids, ordered by lipid class and grouped by gestational age (Fig. 1C). The heatmap shows normalized sphingolipid abundances across individual placental samples. Distinct shifts in the relative abundance of multiple ceramide species and sphingosine, but not sphingomyelin species, were observed between the two gestational time points. To assess which individual sphingolipid species differed significantly between both gestational groups, a volcano plot was generated using a Wilcoxon test (Fig. 1D). The *P*-values and fold changes are reported in Table 3. Long chain ceramides (Cer14:0, Cer16:0, Cer18:0 and Cer18:1) were significantly more abundant in placentae collected at 5–9 weeks of gestation compared to 10–15 weeks of gestation, while content of very long chain ceramides (Cer22:0, Cer24:0, Cer24:1) did not change with advancing gestation (Fig. 1D), which was reflected in the absence of a difference in total Cer content between 5–9 and 10–15 weeks placentae (Fig. 1E). To assess the contribution of de novo synthesis pathway of ceramide to the temporal changes in ceramide levels we interrogated two de novo intermediates, namely sphinganine (Sa) and dihydroceramide (DiHyCers) (18). Sa levels were significantly greater in placental samples from 5–9 weeks than 10–15 weeks of gestation (Table 3). Total DiHyCers did, however, not change with advancing gestation (Fig. 1F). These findings suggest an increased condensation of palmitoyl-CoA and serine to sphinganine but no major changes in its N-acetylation by ceramide synthases (32, 33) to DiHyCers during the early first trimester, when pO_2_ levels are low. Cer levels are further regulated by specific sphingolipid regulatory enzymes (18). Acid (ASAH1) and alkaline (ACER2) ceramidases break down Cer into sphingosine (So), while sphingomyelin phosphodiesterase 1 (SMPD1) converts sphingomyelin into ceramide. Thus, we investigated the expression of all three enzymes across the first trimester of placental development. Total placental SM content remained constant during the first trimester of gestation (Fig. 1G), and no significant change in placental SMPD1 protein expression was noted during the first trimester of gestation (Fig. 2A). This suggests that SM breakdown via SMPD1 did not contribute to long-chain Cer buildup during early placentation (5–9 weeks). We next investigated the temporal expression of both ACER2 (34) and ASAH1 (35). ACER2 (Fig. 2B) and ASAH1 (Fig. 2C, D) displayed differing expression patterns during the first trimester of gestation. ASAH1 expression was significantly lower in placentae from 5 to 9 weeks of gestation compared to 10–15 weeks of gestation, while ACER2 expression remained unchanged. ASAH1 is synthetized in the endoplasmic reticulum as a precursor protein of approximately 55 kDa. The ASAH1 precursor then traffics to the lysosomal compartment where it undergoes enzymatic endocleavage. The bioactive form of ASAH1 is a heterodimer consisting of a non-glycosylated alpha unit of 13 kDa and a glycosylated beta unit of 40 kDa (36). WB analysis for ASAH1 revealed that specifically the 40 kDa glycosylated β-subunit protein increased in placentae collected at 10–15 weeks of gestation compared to those at 5–9 weeks of gestation (Fig. 2D). Together, these findings suggest that changes in long-chain Cer levels vary with the physiological pO_2_ environment of the developing placentae, most likely due to decreased ASAH1 expression.

Sphingosine (So) is the product of the breakdown of Cer by ASAH1 that can be phosphorylated by sphingosine kinases (SPHK) 1 and 2 into sphingosine-1-phosphate (So-1-P) (18). Sphingolipid analyses revealed greater So levels in placentae at 5–9 weeks of gestation compared to 10–15 weeks of gestation, while So-1-P levels did not change during this gestational window (Fig. 1D, Table 3). *SPHK1* mRNA and protein expression were significantly lower in placentae at 5–9 weeks than 10–15 weeks of gestation (Fig. 2E, F), while SPHK2 levels remained constant during the first trimester of gestation (Fig. 2F). This suggests that the So increase during the low oxygen stage of placental development is likely due to reduced SPKH1 expression, while SPHK2 maintains the So-1-P levels.

### Altered ceramide and so-1-P levels in placental *Phd2* knockout mice

**Fig. 3:**
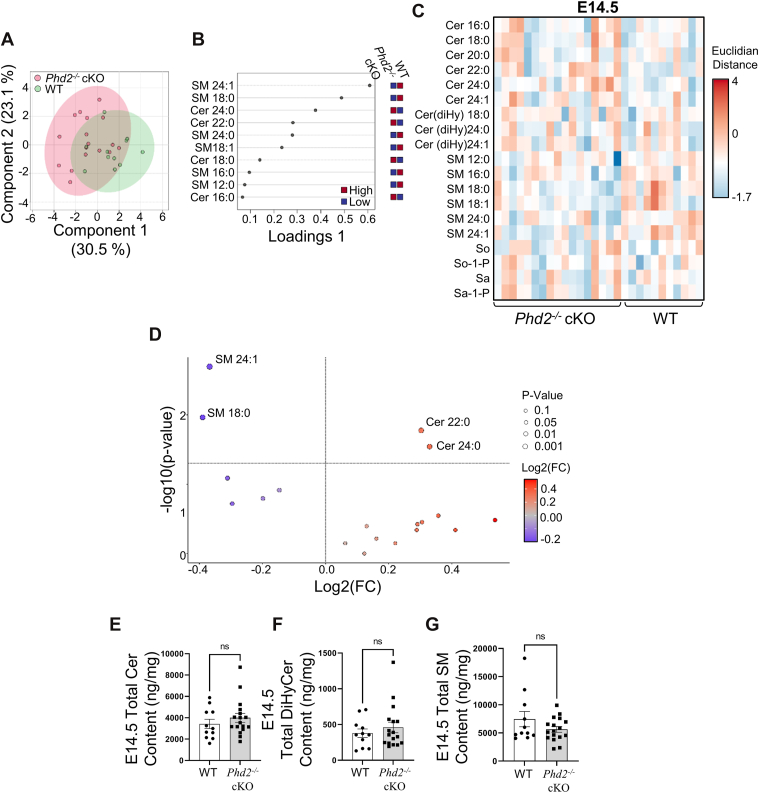
Loss of placental *Phd2* at E14.5 murine pregnancy alters placental sphingolipid metabolism. A: Sparse Partial Least Discriminant Analysis (sPLS-DA) score plot showing the separation in sphingolipids between WT (green) and Phd2^−/−^ cKO (red) murine placental tissue at E14.5. The colored circles represent the 95% confidence regions. B: Loading plots from sPLS-DA showing the top discriminating sphingolipid species contributing to the WT and Phd2^−/−^ cKO group separation. C: Heatmap of sphingolipid species abundance in WT and Phd2^−/−^ cKO placental samples at E14.5, displayed without clustering. Each column represents an individual placenta. Scaled abundance is color-coded from low (blue) to high (red). D: Volcano plot of sphingolipid species abundance in Phd2^−/−^ cKO versus WT placentae at E14.5. Reduced abundance was depicted in blue, while increased abundance is depicted in orange. Statistical significance was evaluated using a Wilcoxon-test and fold-change threshold was set higher than 1.15. Only significant species (*P* > 0.05) were labelled. E – F: scatter plot for (E) total ceramide (Cer), (F) total dihydroceramide (DiHyCer) and (G) total sphingomyelin (SM) content in murine placental samples from WT and Phd2^−/−^ cKO mice at E14.5 (∗: *P* < 0.05, ∗∗: *P* < 0.01, non-parametric Mann–Whitney test). E14.5 samples: n = 4 WT litters, 8 placentae, and n = 4 Phd2^−/−^ cKO litters, 8 placentae.

Having established that long-chain Cer and So content changes during early human placentation, we investigated whether a low oxygen environment was causative of these changes. We employed *Phd2*^*−/−*^ cKO mice, which have the *Phd2* gene conditionally deleted in the placenta, resulting in chronic placental hypoxia as previously shown by hypoxyprobe™ (pimonidazole hydrochloride) immunohistochemistry and heightened HIF1A expression (27, 37). Sphingolipid MS/MS analyses were performed at E14.5 and 17.5 of gestation, corresponding to two important milestones of murine placental development. E14.5 marks the completion of spiral artery remodeling and represents the time point when the murine placenta becomes fully functional. At E17.5 (one day before delivery), the placenta is fully mature and has reached its maximal size and efficiency (38). Consistent with the findings in the human placenta during early (5–9 weeks) gestation (Fig. 1), sPLS-DA revealed partial separation between WT and *Phd2*^*−/−*^ cKO samples at E14.5 (Fig. 3A). The corresponding VIP plot highlighted various individual sphingomyelin (SM12:0, SM16:0, SM18:0, SM18:1, SM24:0, and SM24:1) as well as long chain and very long-chain ceramide (Cer16:0, Cer18:0, Cer22:0, Cer24:0) species as major contributors to the observed group separation (Fig. 3B). A heatmap of normalized values displayed compositional shifts across multiple sphingolipid classes in individual placentae (Fig. 3C). Volcano plot analysis (and Wilcoxon test) identified SM18:0, SM 24:1, and Cer22:0 and Cer24:0 as the most differentially expressed sphingolipid species between the genotypes at this stage (Fig. 3D). The *P*-value and fold change of individual sphingolipids was determined from the corresponding genotype group shown in Table 4. Very long chain Cer22:0 and Cer24:0 were increased in placentae of E14.5 *Phd2*^*−/−*^ cKO mice compared to WT mice, while sphingomyelin species SM18:0 and SM24:1 were decreased in placentae of E14.5 *Phd2*^*−/−*^ cKO mice (Fig. 3D, Table 4).

To determine whether changes in individual ceramide and sphingomyelin species translated into broader alterations in overall sphingolipid class abundance, we quantified total Cer (Fig. 3E), total DiHyCer (Fig. 3F), and total SM (Fig. 3G) in E14.5 placentae. Despite the differences observed at the individual species level, these class-wide measurements revealed no significant differences between *Phd2*^*−/−*^ cKO and WT mice, indicating that the observed ceramide accumulation in placentae of E14.5 *Phd2*^*−/−*^ cKO mice is limited to specific Cer species rather than a generalized increase in all Cer species.

**Fig. 4:**
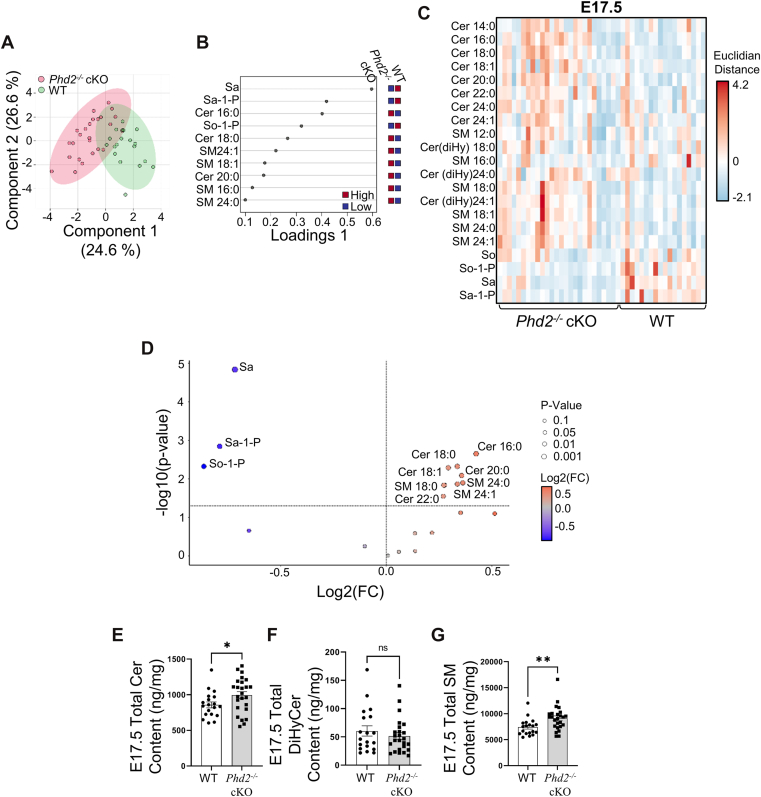
Loss of placental *Phd2* at E17.5 murine pregnancy leads to major changes in placental sphingolipids. A: Sparse Partial Least Discriminant Analysis (sPLS-DA) score plot showing the separation in sphingolipids between WT (green) and Phd2^−/−^ cKO (red) murine placental tissue at E17.5. The colored circles represent the 95% confidence regions. B, Loading plots from sPLS-DA showing the top discriminating sphingolipid species contributing to the WT and Phd2^−/−^ cKO group separation. (C: Heatmap of sphingolipid species abundance in WT and Phd2^−/−^ cKO placental samples at E17.5, displayed without clustering. Each column represents an individual placenta. Scaled abundance is color-coded from low (blue) to high (red). D, Volcano plot of sphingolipid species abundance in Phd2^−/−^ cKO versus WT placentae at E17.5. Reduced abundance was depicted in blue, while increased abundance is depicted in orange. Statistical significance was evaluated using a Wilcoxon-test and fold-change threshold was set higher than 1.15. Only significant species (*P* > 0.05) were labelled. E – G: Scatter plot for (E) total ceramide (Cer), (F) total dihydroceramide (DiHyCer) and (G) total sphingomyelin (SM) content in murine placental samples from WT and Phd2^−/−^ cKO mice at E17.5 (∗: *P* < 0.05, ∗∗: *P* < 0.01, non-parametric Mann–Whitney test). E17.5 samples: n = 5 WT litters, 19 placentae, and n = 5 Phd2^−/−^ cKO litters, 25 placentae.

At E17.5, sPLS-DA showed a clearer separation between WT and *Phd2*^*−/−*^ cKO placentae, with increased variance explained by Component 1 (24.6%) (Fig. 4A). VIP analysis identified Sa, Sa-1-P, So-1-P, Cer16:0, Cer18:0, Cer20:0, and SM18:0 as the top discriminatory sphingolipid species at this gestational stage (Fig. 4B). The sphingolipid heatmap at E17.5 shows broader compositional divergence than observed at E14.5 (Fig. 4C). Volcano plot analysis (and Wilcoxon test) demonstrated that Sa, Sa-1-P, So-1-P were significantly decreased in the *Phd2*^*−/−*^ cKO placentae compared to WT placentae while several Cer and SM species were significantly more abundant in the *Phd2*^*−/−*^ cKO placentae than the WT placentae at this gestational stage (Fig. 4D, Table 5). The *P*-value and fold change of individual sphingolipids were determined from the corresponding genotype group means shown in Table 5. In contrast to the E14.5 stage, the sphingolipid profile at E17.5 revealed broader class-wide alterations. Specifically, the cumulative increase in several individual Cer (Cer16:0, Cer18:0, Cer20:0 and Cer22:0) and SM (SM18:0, SM22:0, SM24:0 and SM24:1) species in *Phd2*^−/−^ cKO placentae (Fig. 4C, Table 5) associated with a significant elevation in total Cer and total SM content (Fig. 4E, G). These findings suggest that the changes in specific sphingolipid species at E17.5 were sufficient in magnitude to shift the overall lipid class abundance. In comparison, total DiHyCer levels remained unchanged between both mouse genotypes (Fig. 4F), suggesting that de novo synthesis of ceramide was not markedly affected by the loss *Phd2* and ensuing hypoxia at this later gestational stage.

**Fig. 5:**
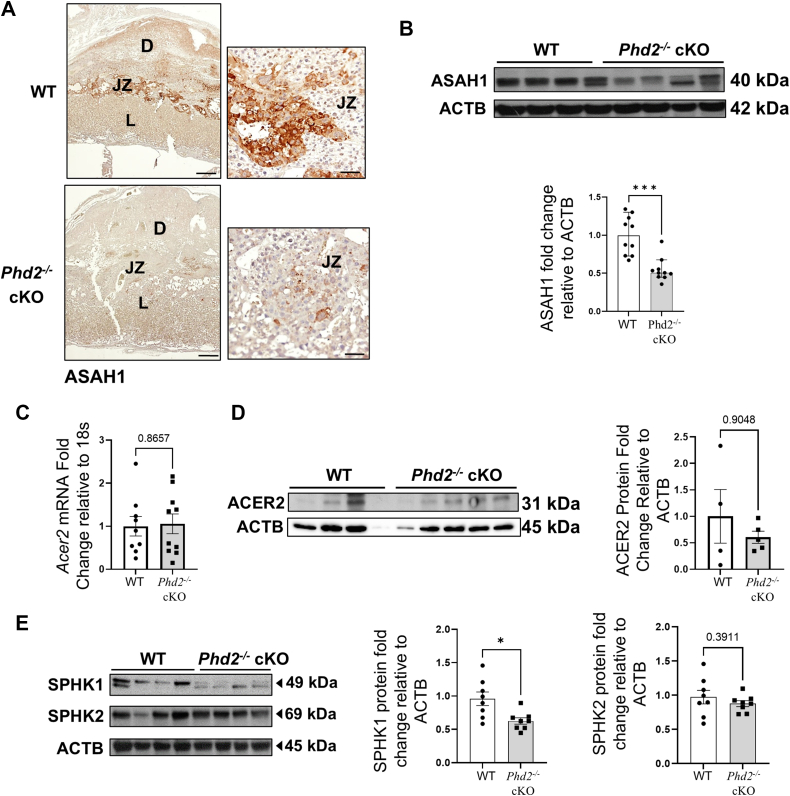
Expression of sphingolipid regulatory enzymes is disrupted upon loss of placental *Phd2* during murine pregnancy. A: Representative IHC staining for ASAH1 in WT and Phd2^−/−^ cKO placental sections at E14.5 (similar results were obtained in 3 separate WT and Phd2^−/−^ cKO placentae, respectively). D: decidua, L, labyrinthine, JZ junctional zone, scale bars = 500 μm. B: representative immunoblot (top panel) and associated densitometry (bottom panel) for ASAH1 in WT and Phd2^−/−^ cKO murine placental samples collected at E14.5 (∗∗∗: *P* < 0.001, non-parametric Mann-Whitney test; n = 4 WT litters, 10 placentae, and n = 4 Phd2^−/−^ cKO litters, 10 placentae). C: quantitative PCR for *Acer2* mRNA in WT and Phd2^−/−^ cKO murine placental samples collected at E14.5 (no statistical differences, unpaired Student’s t test; n = 4 WT litters, 12 placentae, and n = 5 Phd2^−/−^ cKO litters, 13 placentae). D: representative immunoblot (left panel) and associated densitometry (right panel) for ACER2 in WT and Phd2^−/−^ cKO murine placental samples collected at E14.5 (no statistical differences, non-parametric Mann-Whitney test; n = 4 WT litters, 4 placentae, and n = 4 Phd2^−/−^ cKO litters, 5 placentae). E: representative immunoblots (left panel) and associated densitometry (right panel) for SPHK1 and SPHK2 in WT and Phd2^−/−^ cKO placental samples collected at E17.5 (∗: *P* < 0.05, unpaired Student’s t test; n = 4 WT litters, 8 placentae, and n = 4 Phd2^−/−^ cKO litters, 8 placentae). ACTB was used as loading control. ns = nonsignificant.

IHC staining for ASAH1 at E14.5 showed strong positive immunoreactivity in the junctional zone (JZ) and low signal in the labyrinthine (L) and decidual (D) layers of WT placentae (Fig. 5A). Upon deletion of *Phd2* in the JZ layer (27), ASAH1 expression in the JZ was markedly reduced in *Phd2*^*−/−*^ cKO compared to WT placentae (Fig. 5A). These IHC observations were confirmed by immunoblotting, which demonstrated a significant reduction in ASAH1 protein expression in E14.5 *Phd2*^*−/−*^ cKO compared to WT placentae (Fig. 5B). Deletion of *Phd2* in the JZ layer did not affect placental *Acer2* mRNA (Fig. 5C) and protein (Fig. 5D) expression levels at E14.5. In line with its reported temporal expression in mice (39), no placental *Acer2* mRNA and protein were detected at E17.5 (not shown). Sphingolipid MS/MS analyses revealed no significant differences in So content between WT and *Phd2*^*−/−*^ cKO placentae at E14.5 and 17.5 of gestation (Figs. 3D and 4D). Although So-1-P content was not different between E14.5 *Phd2*^*−/−*^ cKO and WT placentae, it was significantly reduced in *Phd2*^*−/−*^ cKO placentae compared to WT placentae at E17.5 (Fig. 4D, Table 5). Consistent with the human placenta, immunoblotting for SPHK1 at E17.5 revealed a significant reduction of SPHK1 expression in *Phd2*^*−/−*^ cKO versus WT placentae, while SPHK2 expression was unchanged (Fig. 5E). Overall, our findings suggest that the chronic hypoxic environment of the *Phd2*^*−/−*^ cKO placenta enhances Cer buildup and diminishes So-1-P levels due to reduced expression of ASAH1 and SPHK1, respectively.

### Low oxygen promotes mitochondrial fission events during early human placental development

**Fig. 6:**
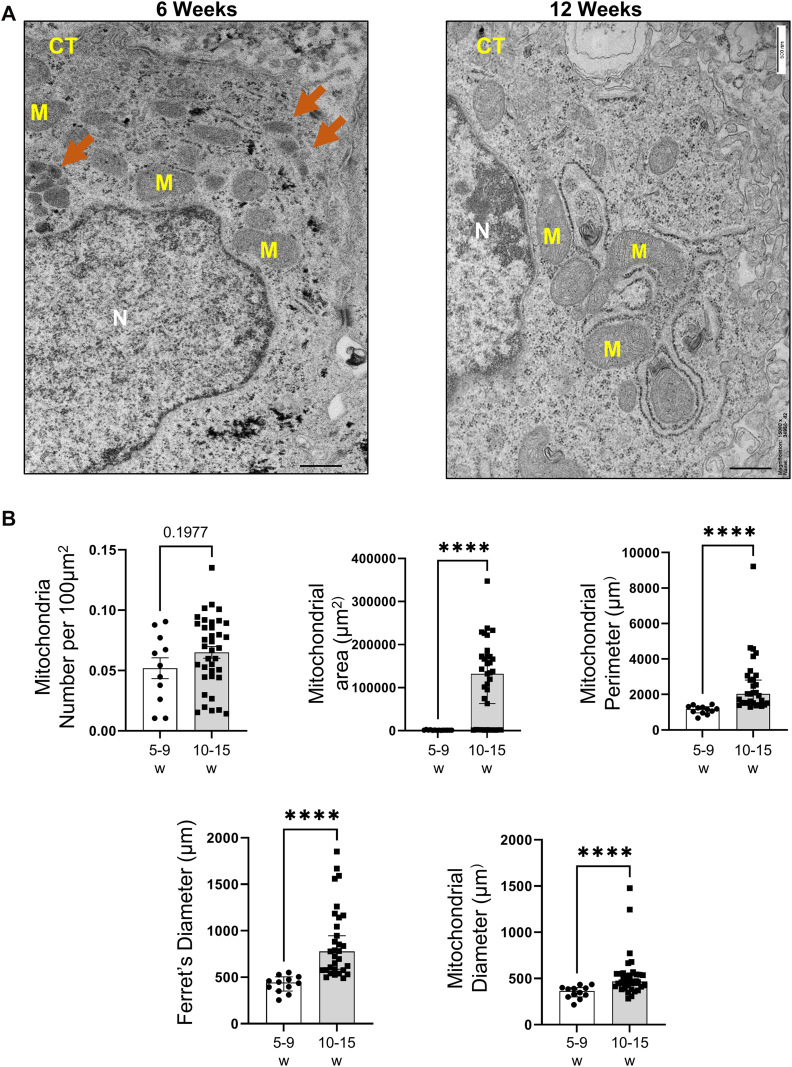
Mitochondrial dynamics is tilted toward fission during early human placental development. *A*, representative TEM images of sections from human placental tissue collected between 5 and 15 weeks of gestation (scale bars = 500 nm). Orange arrows indicate fragmented mitochondria. M, mitochondria; CT, cytotrophoblast, N, nucleus. *B*, mtochondrial morphometric analysis of cytotrophoblast cells from human placental tissue collected between 5 and 15 weeks of gestation (∗∗∗∗*P* < 0.0001, non-parametric Mann-Whitney test, minimum of 4 fields per n, n = 3 human samples from 5 to 9 weeks, 14 fields; n = 3 human samples from 10 to 15 weeks, 30 fields).

Recently, we reported that hypoxia-induced upregulation of long-chain Cer levels in the human placenta (35) and human trophoblastic cells (20) led to a shift in trophoblast mitochondrial homeostasis towards fission (20, 23). To confirm these observations, we conducted ultrastructural TEM analysis to monitor trophoblast mitochondrial morphology in placentae during the first trimester of gestation. The ultrastructural analysis demonstrated significantly smaller mitochondria (reduced surface area, diameter and perimeter) in trophoblast cells of placental sections from 5-9 weeks compared to 10–15 weeks of gestation (Fig. 6A, B). The Ferret diameter was also significantly lower in mitochondria of trophoblasts at 5–9 weeks of gestation (Fig. 6B), indicative of increased fragmentation (26). Interestingly, the number of mitochondria per trophoblast tissue area did not change between 5-9 and 10–15 weeks of gestation (Fig. 6B). Together, these data suggest increased mitochondrial fission in human trophoblast cells during early placentation when physiological pO_2_ is low and long-chain Cer levels are increased.

### Low oxygen promotes mitochondrial fission in placental *Phd2* knockout mice

**Fig. 7:**
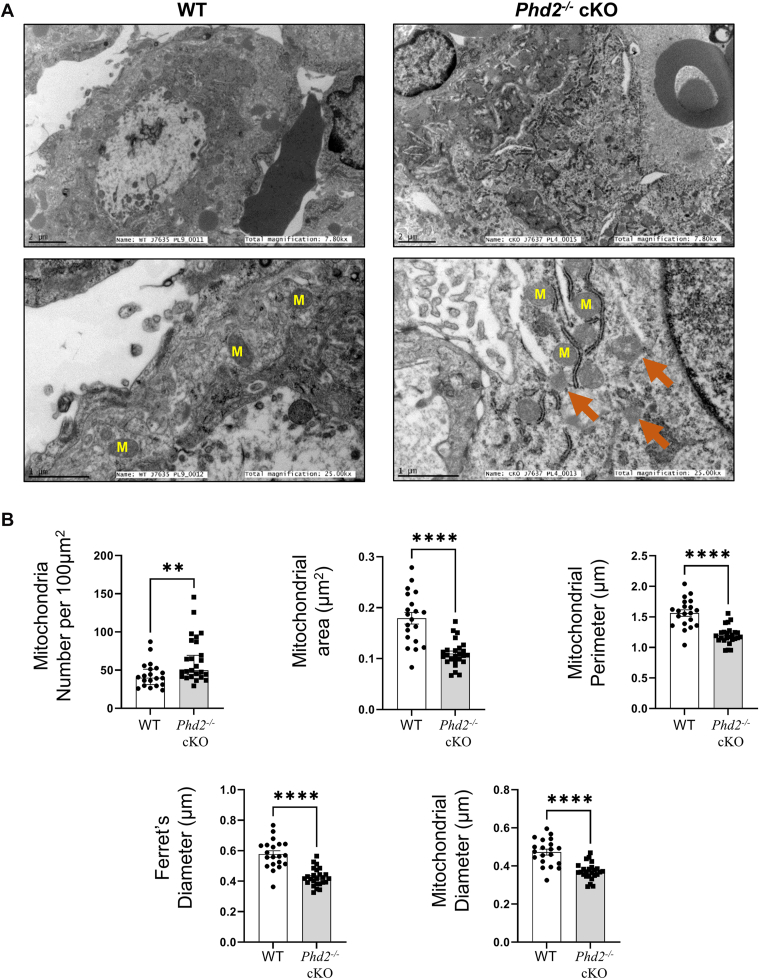
Placental loss of *Phd2* leads to increased mitochondrial fission in the labyrinth. A: representative TEM images from sections of WT and *Phd2*^*−/−*^ cKO placental samples collected at E14.5 (top panel: scale bars = 2 μm; bottom panel: scale bars = 1 μm). Orange arrows indicate fragmented mitochondria. M, mitochondria. B: mitochondrial morphometric analysis of labyrinth layer from WT and *Phd2*^*−/−*^ cKO murine placental samples collected at E14.5 (∗∗*P* < 0.01, ∗∗∗∗*P* < 0.0001, unpaired Student’s *t* test, relative to mitochondria of WT group, minimum of 4 fields per n, n = 3 litters from WT pregnant dams, 20 fields, n = 3 litters from *Phd2*^*−/−*^ cKO placentae, 25 fields).

**Fig. 8:**
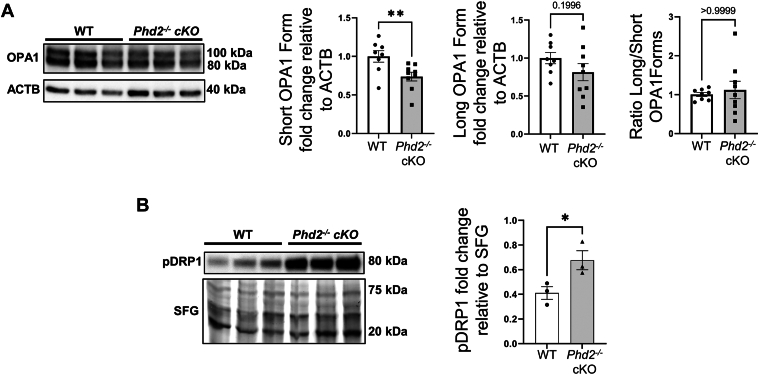
Placental loss of *Phd2* upregulates expression of pDRP1 while decreasing that of OPA1. A, B: Representative immunoblots (left panel) for mitochondrial fusion marker OPA1 (A) and fission marker pDRP1 (B) and associated densitometry (right panels) in WT and Phd2^−/−^ cKO placental samples collected at E14.5 (∗: *P* < 0.05, ∗∗: *P* < 0.01, unpaired Student’s t test, n = 5 WT litters, 10 placentae, and n = 5 Phd2^−/−^ cKO, 10 placentae).

Previously, we have shown that placental Cer content is increased in preeclampsia due to a persistent state of placental hypoxia (35), leading to heightened mitochondrial fission events (20, 23). As Cer levels were also increased in hypoxic *Phd2*^*−/−*^ *cKO* placentae (Figs. 3D and 4D), we investigated whether placental mitochondrial homeostasis was altered. Ultrastructural analyses of E14.5 *Phd2*^*−/−*^ cKO placental sections revealed an increase in the number of mitochondria relative to WT E14.5 placentae (Fig. 7A, B). Compared to E14.5 WT mice, mitochondria from E14.5 *Phd2*^*−/−*^ cKO placental sections were significantly smaller, exhibiting a reduced surface area, perimeter and diameter (Fig. 7B). The reduced Ferret diameter suggests that mitochondria from E14.5 *Phd2*^*−/−*^ cKO placentae undergo more fragmentation than those of E14.5 WT placentae (Fig. 7B). In support of increased mitochondrial fission (20), placentae from E14.5 *Phd2*^*−/−*^ cKO also exhibited increased tethering of mitochondria to ER structures (Fig. 7A). To corroborate the ultrastructural results, we separately quantified the long (100 kDa) and short (80 kDa) isoforms of the mitochondrial fusion marker OPA1 (40). L-OPA1 isoforms promote fusion of the inner mitochondrial membrane (40, 41). R Under cellular stress L-OPA1 is cleaved to fusion-inactive S-OPA1 to enhance survival (40, 41, 42). Western blot analysis revealed a significant decrease in the short isoform, while the long isoform and the short/long ratio remained unchanged in E14.5 Phd2^−/−^ cKO versus WT placentae (Fig. 8A), suggesting no change in fusion events. In parallel, levels of the mitochondrial fission marker pDRP1 (20, 26) were increased in cKO placentae (Fig. 8B). Taken together, the data show that in the chronic hypoxic environment of the *Phd2*^*−/−*^ cKO placenta (27, 37) mitochondrial dynamics is tilted towards fission, like human placentae at the early trimester of gestation (Fig. 6) when the physiological pO2 is low.

## Discussion

Herein, we demonstrate that a low oxygen environment, whether physiological, experienced by the human placenta during early gestation or induced in a murine placental conditional knockout of *Phd2*, leads to a unique placental sphingolipid signature that promotes mitochondrial fission in trophoblast cells. In the human placentae, we found elevated long-chain Cer and So levels during the early physiological low-oxygenated environment (5–9 weeks) compared to 10–15 weeks of gestation when oxygenation increases. This was likely due to a reduced expression of ASAH1, a lipid hydrolase that breaks down Cer to sphingosine (So) and fatty acids, and SPHK1, an enzyme that phosphorylates So to So-1-P. In contrast, ACER2, a ceramidase that breaks down Cer under alkaline conditions (39), was unchanged in the placenta during early gestation, suggesting no direct role in the increase of long-chain Cer at this gestational window. Similar observations regarding long-chain Cer accumulation and ASAH1 expression were made in the chronic hypoxic placentae of *Phd2*^*−/−*^ cKO mice that mimics preeclampsia (27, 37). Additionally, we found that low oxygen-induced long-chain Cer buildup in human placentae from early gestation (5–9 weeks) and in *Phd2*^*−/−*^ cKO murine placentae was accompanied by trophoblast mitochondrial dynamics being tilted towards fission, as evidenced by the presence of smaller and more globular mitochondria, and increased pDRP1. Collectively, these findings highlight the intricate interplay between pO_2_, sphingolipid metabolism and mitochondrial dynamics, and how an imbalance in this interaction may contribute to placental dysfunction in pregnancy-related disorders such as preeclampsia.

Our sphingolipid analyses demonstrated a buildup of long-chain Cer species (18) in early human placental development when oxygen tension is low (2, 4). Importantly, not all ceramide species exert the same biological effects. Previous studies have demonstrated that C16-Cer is generally pro-apoptotic, promoting mitochondrial dysfunction and cell death, whereas very-long-chain ceramides such as C24-Cer can have protective or pro-survival effects. These opposing roles may help explain the distinct mitochondrial responses observed across developmental stages in our study (43). Previously, we have shown that long chain Cer accumulates primarily in the trophoblast layers of the preeclamptic (hypoxic) placenta due to diminished ASAH1 expression (35, 40). We assume that the long-chain Cer buildup during the low-oxygen environment of early human placentation occurs in the same placental layers, likely via a similar mechanism. Indeed, heightened placental long-chain Cer content was accompanied by a reduction in ASAH1 levels. In contrast, ACER2 expression was unchanged in early placentation, arguing against a role in the long-chain Cer buildup occurring at that phase. Spatial expression of the ceramidases was not investigated. It is plausible that the hydrolases are expressed in different placental compartments, i.e., ASAH1 in trophoblasts (35) versus ACER2 in endothelial cells (34, 39), which could explain the different temporal expression patterns. Surprisingly, the reduction in ASAH1 protein did not lead to less sphingosine formation. In contrast, placental sphingosine content was increased most likely due to diminished SPHK1 expression and subsequent reduced phosphorylation of So to So-1-P.

Decreased acylation of sphingosine via ceramide synthase in the salvage pathway or increased conversion of So-1-P into sphingosine by So-1-P phosphatases could also contribute to greater sphingosine levels, though these pathways were not investigated in the current study. Although placental sphinganine (Sa) content was increased, DiHyCer levels were not, suggesting that long-chain Cer accumulation during the low-oxygenated stage of human placental development is not due to heightened de novo synthesis (17). Enhanced formation of Cer from sphingomyelin degradation by sphingomyelinases is another possibility, but stable sphingomyelin and lysosomal SMPD1 levels during the first trimester of placentation argue against a major contribution of the SM degradation pathway to the long-chain Cer buildup. We did not measure glycosylceramides as we previously showed that their breakdown did not contribute to the elevated Cer levels of preeclamptic (hypoxic) placentae (35). The hypoxic environment in placentae of *Phd2*^*−/−*^ cKO mice (27) also led to an increase in long-chain Cer content and decrease in ASAH1 expression, mimicking our reported findings for human preeclampsia (35, 44). In support of this, we observed a marked decrease in basal levels of very long-chain ceramides (C22 and C24) between E14.5 and E17.5 in wild-type placentae, which may reflect physiological adaptations in ceramide metabolism driven by gestational age or oxygen availability. Previously, we have shown that exposure of placental explants/cells to hypoxia/oxidative stress causes similar changes in long-chain Cer content and ASAH1 expression (35), underscoring low oxygen’s direct action on sphingolipid metabolism. In the present study, we also observed a significant reduction in SPHK1 expression and So-1-P content in E17.5 *Phd2*^*−/−*^ cKO placentae, which aligns with previous findings showing that SPHK1 expression and So-1-P levels are downregulated in placentae from preeclamptic patients (22, 45), suggesting that the placental SPHK1/So-1-P pathway is compromised in preeclampsia. Like preeclampsia, placental hypoxia in intrauterine growth restriction (IUGR) has been shown to disrupt sphingolipid metabolism, with increased So levels and decreased Cer content (46). This dysregulation of sphingolipid metabolism in IUGR, characterized by augmented trophoblast cell death, parallels the changes observed in our study and further supports the role of altered sphingolipid metabolism contributing to placental dysfunction under hypoxic conditions (46).

In the placenta, a balance between Cer and So-1-P functions as a critical rheostat that governs trophoblast cell fate under varying oxygen conditions (18, 35). Placental ceramidases ASAH1 and ACER2 (34, 35, 39, 47) regulate this axis by converting ceramide into sphingosine, which is then phosphorylated by placental sphingosine kinases (SPHK1/2) to So-1-P (48). This metabolic rheostat is pivotal as ceramide and sphingosine accumulation can trigger trophoblast apoptosis or autophagy (18, 49), whereas So-1-P promotes cell survival, proliferation, and angiogenesis (18, 50). Under physiological low-oxygen conditions of early placental development**,** ACER2 and SPHK2 activities maintain a favorable amount of So-1-P that supports trophoblast outgrowth and placental vascular development. Severe hypoxia (1% O_2_) has been reported to rapidly induce SPHK1 expression (via HIF1A), boosting So-1-P production (51), whereas more moderate, physiologic *h*ypoxia (∼8% O_2_) has little effect (51). This HIF1–SPHK1 coupling suggests that sphingolipid flux is an adaptive mechanism to counteract oxygen deprivation, promoting angiogenesis and survival in the developing placenta (51). In contrast, pathological hypoxia in disorders like preeclampsia tilts the Cer/So-1-P rheostat toward a pro-death state. Preeclamptic placentae exhibit lower SPHK1 expression and So-1-P levels, concomitant with down-regulation of pro-angiogenic So-1-P receptors (So-1-P R1/3) and up-regulation of the anti-angiogenic So-1-P R2 (51). This dysregulation tilts trophoblasts toward apoptotic cell death and impairs placental angiogenesis, contributing to the characteristic shallow invasion and vascular insufficiency typical of preeclampsia. Herein, similar changes in SPHK1 expression and So-1-P content were noted in *Phd*^*−/−*^ cKO mice that display abnormal placentation, impaired remodeling of the uterine spiral arteries, and fetal growth restriction (27). ACER2 is essential for placental vascular integrity as ACER2-deficient mice fail to maintain normal placental vasculature due to disrupted sphingolipid homeostasis (39). Mechanistic studies indicate that HIF2A upregulates ACER2 expression, thereby accelerating ceramide catabolism to favor So and So-1-P production under stress (52). Thus, loss or insufficient induction of ACER2 under hypoxic stress would impair the placenta’s ability to dispose of ceramide, exacerbating cell death signals. In the present study, we observed unchanged ACER2 expression during early gestation and under sustained placental hypoxia (*Phd2*^−/−^ cKO mice). Previously, we found that only HIF1A, but not HIF2A, is upregulated in placentae of preeclamptic pregnancies (13) and *Phd2*^*−/−*^ cKO mice (27) although others have implicated HIF2A (53, 54). The ASAH1 promoter does not contain any hypoxic response binding sites, suggesting no direct regulation by HIF1/2 (35). However, the promoter binds the E2F4 transcription repressor, which is upregulated in preeclampsia via the activated HIF1A/TGFB3 axis (13, 35). This makes ASAH1 a target under severe placental hypoxia and, indeed, under oxidative conditions, ASAH1 is downregulated in trophoblastic JEG cells, resulting in elevated Cer (35). Intrauterine growth restriction (IUGR) placentas also show a sphingolipid imbalance**,** *e.g*. elevated So due to heightened ceramide breakdown (via ASAH1) coupled with reduced SPHK1 activity, which is linked to increased trophoblast cell death (46). In summary, the Cer/So-1-P rheostat, governed by enzymes like ASAH1, ACER2 and SPHK1/2, is a crucial determinant of placental development under both normoxic and hypoxic environments. Its proper regulation promotes trophoblast proliferation and placental angiogenesis, whereas its dysregulation under pathological hypoxia directly contributes to placental disorders such as preeclampsia and IUGR.

The significance of SPHK1 and 2 in embryonic development is highlighted by findings that double knockout of these enzymes leads to embryonic lethality in mice, primarily due to defects in neural and vascular formation as well as decidualization (55, 56). Interestingly, an individual knockout of either SPHK gene does not result in notable abnormalities, suggesting that these two isoenzymes have overlapping and complementary functions in normal physiological processes (56, 57). Although both SPHK1 and SPHK2 catalyze the production of So-1-P, they seem to operate through different mechanisms; for example, SPHK1 often acts as a strong anti-apoptotic factor and mitogenic stimulator, while increased SPHK2 expression induces cell death in specific contexts (57). This distinction highlights the complexity of sphingolipid signaling pathways during development. Understanding the specific roles of SPHK1 and SPHK2 is crucial for elucidating their contributions to placental development, particularly in the context of oxygen changes. Herein, we found reduced SPHK1 protein expression during the hypoxic stage of human placentation and in chronic hypoxic *Phd2*^*−/−*^ cKO placentae. Placental SPHK2 expression appeared unaffected by intrauterine oxygen fluctuations, suggesting that oxygen-induced placental So-1-P alterations are mainly due to changes in SPHK1 expression. The SPHK1 promoter contains a HIF1A binding element (58) and HIF1 is upregulated in human placentae at 5–9 weeks of gestation and in early-onset preeclampsia (3, 13, 59). Contrary to our findings of reduced placental SPHK1 expression, studies using JAR cells and chorionic villous explants have shown upregulation of SPHK1 expression by severe hypoxia via HIF1A activation (18, 51). However, in line with our observations, SPHK1 expression was markedly decreased in pregnancies complicated by IUGR, through impaired ALK1/SMAD1 signaling caused by HIF1-mediated aberrant TGFβ signaling (46). Evidently, the exact mechanism(s) by which HIF1 regulates SPHK1 expression during placental development and preeclampsia warrants further investigation. Long chain ceramides and sphingosine are generally associated with increased cellular stress, cell cycle arrest and canonical cell death modes such as apoptosis, necroptosis and autophagy (18). Conversely, So-1-P produced by SPHK1/2 is linked to cell survival processes such as protective autophagy, cell migration and invasion and cytoskeleton rearrangement (18). We speculate that under physiological conditions, such as transient hypoxia experienced during early gestation, regulation of SPHK activity and So-1-P product may facilitate appropriate placental adaptation and growth. In contrast, under pathological conditions that are characterized by sustained hypoxia, like preeclampsia, disruptions in sphingolipid metabolism may lead to impaired placental function and adverse outcomes for both mother and fetus. Interestingly, while SPHK2 expression remained stable in both early gestation and *Phd2*^*−/−*^ cKO placentae, S1P levels only declined in the latter, suggesting a possible divergence in SPHK2 functional activity between physiological and pathological hypoxia. Thus, the interplay between intrauterine pO_2_, SPHK expression and So-1-P formation is vital for maintaining placental health and could provide insight into potential therapeutic targets for managing conditions associated with abnormal placentation such as preeclampsia.

The implications of sphingolipids in cell fate events have been well-documented in various pathophysiological processes (17, 18, 60). Genetic deficiencies in enzymes involved in sphingolipid metabolism, such as ASAH1 and SMPD1, are associated with lysosomal sphingolipid storage disorders such as Farber disease and Niemann-Pick disease, respectively, emphasizing the importance of maintaining proper sphingolipid balance (61, 62). In pregnancy, we characterized preeclampsia as a new ‘sphingolipid storage’ disorder as placentae from preeclamptic patients accumulate long-chain Cer due to diminished ASAH1 expression (35). In the present study, we capitalized on our murine *Phd2*^*−/−*^ cKO model of early-onset preeclampsia, characterized by placental hypoxia and sustained HIF1A levels (27). We confirmed that placentae of murine *Phd2*^−/−^ cKO pregnant mice exhibited similar alterations in sphingolipid metabolism as reported for preeclampsia in humans, except for sphinganine (35). This comparative approach allowed us not only to assess the potential translational relevance of findings across species but also to enhance our understanding of the complex interplay between oxygen and sphingolipid metabolism in normal placentation and disease.

In human preeclampsia, we reported that mitochondrial long-chain Cer buildup tilted placental mitochondrial dynamics towards mitochondrial fission, with smaller and more fragmented mitochondria, driven in part by increased expression of the Bcl-2 family member BOK (20). Similarly, in the present study, we observed that the chronic hypoxic environment in *Phd2*^−/−^ cKO also led to placental long-chain Cer buildup and a concomitant shift toward mitochondrial fission. Studies conducted with primary mesenchymal cells isolated from human placentae of 5–9 weeks gestation have demonstrated that a physiological low pO_2_ is sufficient to promote mitochondrial fission events in these cells (23). In the present study, we confirmed that human placentae during the early (5–9 weeks) hypoxic stage of gestation exhibit heightened levels of mitochondrial fission. The finding of elevated mitochondrial fission under physiological low pO_2_ highlights potential adaptive mechanisms employed by the placenta during early development to counter the transient accumulation of long-chain Cer. Mounting evidence from other systems supports a direct mechanistic link between Cer and mitochondrial fragmentation. In muscle cells, cardiomyocytes, and trophoblasts, exogenous Cer loading activates the fission GTPase DRP1, triggering rapid mitochondrial fission, an effect that is reversed by DRP1 inhibition (20, 63, 64). In trophoblasts, Cer16:0 has been shown to induce BOK expression at ER–mitochondria contact sites (MAMs), which in turn promotes DRP1 recruitment and mitochondrial fission (20). Recent findings have also shown that DRP1 expression can be upregulated by hypoxia-inducible factor 1-alpha (HIF-1α), linking sustained hypoxic signaling directly to enhanced mitochondrial fission activity (65). Beyond direct mitochondrial signaling, lysosomal dysfunction also contributes to altered mitochondrial dynamics. Dysregulation of enzymes such as SMPD1 and ASAH1 alters lysosomal ceramide content, skewing the balance toward mitochondrial fragmentation (35). For example, increased lysosomal ceramide, whether due to ASM hyperactivity or ASAH1 deficiency, has been shown to enhance DRP1-mediated fission, interfere with mitophagy, and impair mitochondrial homeostasis (66, 67). Additionally, Cer can engage mitochondrial fission factor (MFF) directly, reinforcing DRP1 recruitment to the outer mitochondrial membrane. These inter-organelle signaling pathways highlight Cer as more than a biomarker of cellular stress; it is a potent effector molecule capable of remodeling mitochondrial structure and function (17). Taken together, these findings support the model that placental long-chain ceramide accumulation, under both physiological and pathological hypoxia, can modulate mitochondrial architecture through conserved fission pathways.

Limitations of the study include the absence of 1-deoxysphingolipid quantification, as these species were not part of the targeted lipidomics panel. Given their potential relevance under conditions of altered amino acid metabolism and oxygen tension, their omission may limit a more complete understanding of placental sphingolipid remodeling. Additionally, although we report reduced ASAH1 expression, we did not assess its enzymatic activity directly. The enzymatic activities of SPHK1 and SPHK2 were also not measured, and given their distinct regulatory mechanisms and potential divergence in function under physiological versus pathological hypoxia, this remains an important gap. While our findings suggest a potential link between ceramide accumulation and increased mitochondrial fission, the data remain correlative. To establish causality, future studies should incorporate 1-deoxysphingolipid profiling, functional assays of key enzymes, and direct perturbation of ceramide levels in trophoblasts to delineate the mechanistic relationship between ceramide metabolism and mitochondrial dynamics.

Overall, the present study contributes to our understanding of the interplay between the placental pO_2_-HIF1 axis, sphingolipids and mitochondrial dynamics and their importance for placental development and disease. Additionally, the similarities in molecular sphingolipid signatures between *Phd2*^*−/−*^ cKO pregnant mice and human preeclampsia further underscore the validity of the *Phd2*^−/−^ cKO pregnant mice as a model to study human preeclampsia.

## Data availability

The data presented in this manuscript are available within the manuscript itself. Any additional data related to this study can be shared upon reasonable request. Please contact the corresponding author for access to these datasets.

## Conflict of Interest

The authors declare that they do not have any conflicts of interest with the content of this article.
